# 146. Intact Sense of Taste and Smell During COVID-19 Infection Is Associated with Absence of of SARS-CoV-2 Spike Protein Antibody Responses within 3 Months of Symptomatic Illness

**DOI:** 10.1093/ofid/ofab466.146

**Published:** 2021-12-04

**Authors:** James M Wilson, Sheena Gillani, Robert Bencshop, Josh Poorbaugh, Ajay Nirula, Lin Zhang, Kody Keckler, Kathleen Weber, Ralph Morack, Stephanie Beasley, Jennifer Brothers, Gregory Huhn

**Affiliations:** 1 Rush University Medical Center / Cook County Health System, Chicago, IL; 2 Rush University Medical Center, Chicago, Illinois; 3 Eli Lilly, Indianapolis, Indiana; 4 Cook County Health System, Chicago, Illinois; 5 Cook County Health Systems, Chicago, Illinois; 6 Hektoen Labs, Chicago, Illinois; 7 John H Stroger Jr. Hospital of Cook County, Chicago, Illinois

## Abstract

**Background:**

Although studies show most COVID-19 survivors have post-infection immunity against SARS-CoV-2 that could prevent re-infection, there is still a need to identify the breadth of antibody (Ab) responses associated with clinical phenotypes. We characterized Ab profiles at the estimated peak of Ab diversity among adults with recovered SARS-CoV-2 infections and determined their relationships with clinical factors.

**Methods:**

From April-June 2020, 41 health system employees with PCR-confirmed symptomatic COVID-19 infection enrolled 8-10 weeks after symptom onset. Symptom questionnaires including baseline demographics, COVID-19 symptoms, disease severity, and disease duration were collected and plasma samples were assayed using a custom Luminex Multiplex platform (Figure 1) to measure the antibody response against 20 COVID-19 related antigens (Figure 2). Differences in Ab profile titers among different groups were tested using nonparametric t test and Benjamini-Hochberg adjustment for multiplicity. Associations were considered significant at FDR< 0.05.

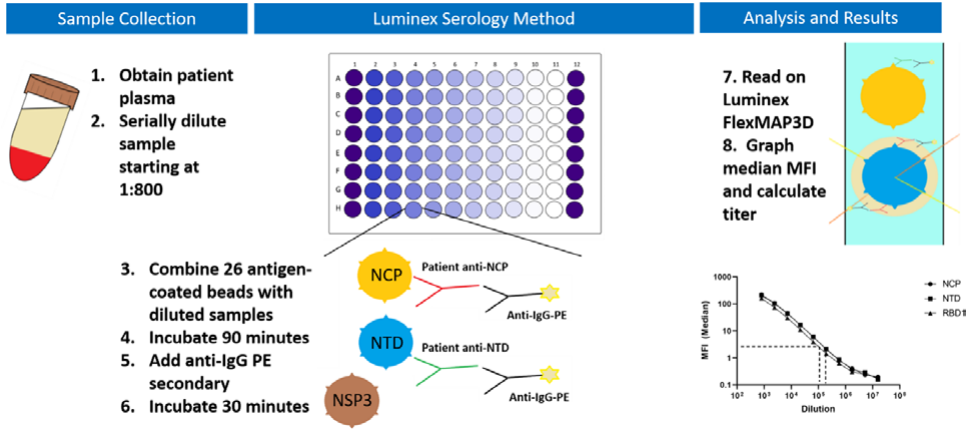

Figure 1: Description of the Luminex Serology Assay

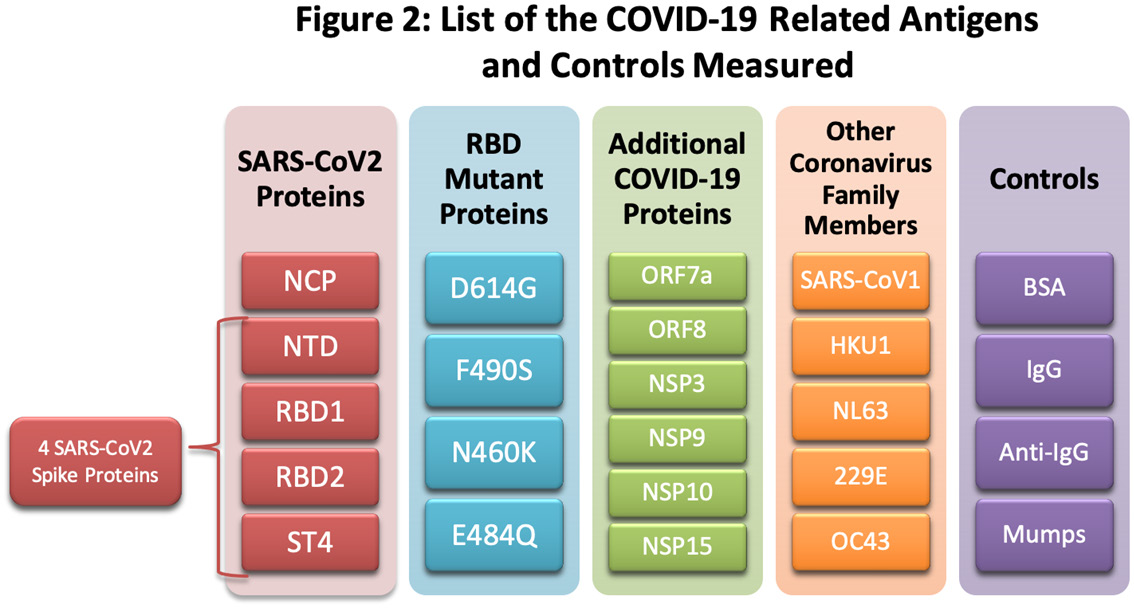

Figure 2: List of the COVID-19 Related Antigens and Controls Measured

**Results:**

Mean age was 48 years (range 27-68), with 51% female, 37% White, 32% Black, 29% Asian, and 17% LatinX. Ab profiles (Figure 3) showed 100% cross-reactivity with related alpha and beta coronavirus, and 95% with SARS-CoV-1. 78% had Abs against SARS-CoV-2 nucleocapsid protein (NCP). However, 29% of patients had no immune response against the four spike protein epitopes. These participants also reported fewer symptoms, including no cases of anosmia/ageusia, suggesting mild illness. Anosmia/ageusia, fever, and cough associated significantly with higher Ab titers (Figure 4).

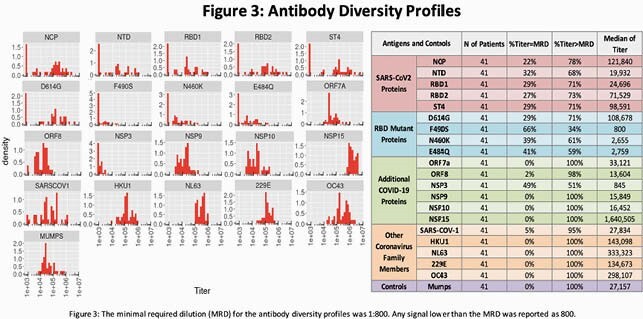

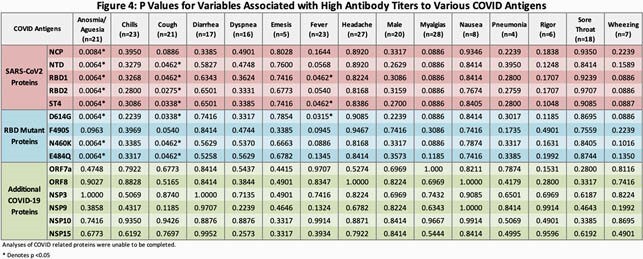

**Conclusion:**

Broad immune responses to various SARS-CoV-2 and related antigens were found among a heterogeneous patient population. However, less than 3 months after symptom onset, protective Ab responses to SARS-CoV-2 spike proteins were not detected in nearly one-third of recovered patients, primarily with mild infection. Intact sense of smell and taste demonstrated the greatest association with loss of seroprotective SARS-CoV-2 Ab responses, which may be clinically useful to predict post-infection immunity. Next steps include comparing the magnitude of Ab responses following full series completion with mRNA vaccination among this cohort.

**Disclosures:**

**Robert Bencshop, PhD**, **Eli Lilly** (Employee) **Josh Poorbaugh, PhD**, **Eli Lilly** (Employee) **Ajay Nirula, MD/PhD**, **Eli Lilly** (Employee, Shareholder) **Lin Zhang, PhD**, **Eli Lilly and Company** (Employee, Shareholder) **Stephanie Beasley, BA**, **Eli Lilly** (Employee)

